# Nursing management of patients dealing with spina bifida: from the prenatal diagnosis to adulthood / nursing intervention for the improvement of the impact of urinary and fecal incontinence on the quality of life of people dealing with spina bifida

**DOI:** 10.1186/s13052-024-01579-z

**Published:** 2024-03-13

**Authors:** Fabiana Calabrese, Antonio Poziello, Gennaro Spiezia, Tiziana Rotunno, Ciro Chervino, Anna Maria Iannicelli

**Affiliations:** 1 Department of Translational Medical Sciences, “Federico II” University, Via Pansini 5, 80131 Naples, Italy; 2 CNS Unit of Interventional Neuroradiology, Department of Advanced Biomedical Sciences, “Federico II” University, Naples, Italy; 3 Department of Neurosciences, Science of Reproduction and Odontostomatology, “Federico II” University, Naples, Italy

**Keywords:** Spina Bifida, Urinary incontinence, Fecal incontinence, Constipation, Quality of life, Nursing intervention, Autonomy

## Abstract

**Background:**

Urinary and fecal incontinence in people dealing with spina bifida, has inevitably an influence on the quality of life. In this analysis, the degree of education on how to manage incontinence and retention is studied, as well as the problems those might create and the consequential degree of autonomy and independence reached into the management of those. The main goal is to increase both nursing assistance and the education of the people dealing with spina bifida.

**Methods:**

A multiple-choice questionnaire with open questions, concerning the bowel and bladder management was structured by all the authors and shared by the Google Docs platform among the members of the ASBI (Associazione Spina Bifida Italia) by the secretariat of the association itself. 125 patients affected by Spina Bifida voluntarily decided to participate and complete the questionnaire. The questionnaire didn’t set any limits as regards the age. For minors, its completion was made under the observation of the caregivers who gave their consent. All the authors participated to administration of the questionnaire to minors.

**Results:**

out of 125 participants, 80 were females and 25 males. The questions concerned the level of deambulation (the 35,2% was autonomous, the 30,4% were people who use wheelchairs while the 34,4% is aid-supported), urinary incontinence, with great concern to the self-catheterization technique (the 80,8% claimed to be autonomous in performing self-catheterization, unlike the remaining 19,2%) and the impact of the said incontinence on social life (the 59,2% claimed they do not feel restrained because of their bladder incontinence or retention, unlike the remaining 40,8%). Lastly, we focused on fecal constipation and incontinence (the 57,6% claimed to struggle with incontinence, the 12% claimed they don’t and the 30,4% struggles with both conditions), on the ability of the people dealing with this to intervene to prevent unpleasant situations, in particular by using trans-anal irrigation (the 57,6% doesn’t feel autonomous in performing it).

**Conclusion:**

urinary and fecal incontinence have, of course, an impact on the quality of life of people dealing with spina bifida. Nevertheless, we can observe that it is possible to improve the quality of life of these people, letting them feel confident enough to take part in social activities, through education from an incredibly young age, from 0 up to 25 years old and over, supplied by the medical staff and mostly by the parents (previously educated by the medical staff as well).

**Supplementary Information:**

The online version contains supplementary material available at 10.1186/s13052-024-01579-z.

## Background

The Spina Bifida is a severe congenital malformation that interests the central nervous system; it applies 1 pregnancy every 1000 and it is the most common congenital non-chromosomal flaw [1].

The Spina bifida originates during the embryogenesis and derives from the failed closure of the neural tube, with a consequential split of the spine (bifida), in between the 18th and 28th embryonic day [2]. 

This creates flaws in the vertebral arches, sometimes concerning the spinal cord as well [3]. From the anomalous vertebral fusion and the location of the lesion, it is possible to observe a variety of neurological deficits [4]. Spina Bifida is one of the most complex conditions in the medical field, as it concerns the congenital and evolutionary sphere, as well as the neurological and orthopedic one, the motor and perceptive one, the somatic and bowel one and the psychological and organic one [2]. Dealing with a patient struggling with spina bifida is a multidisciplinary task. In particular, urinary incontinence (UI) and fecal incontinence (FI) are very common in people dealing with spina bifida and they influence the quality of their lives [5]. Different studies have analyzed this phenomenon: it was reported that the 66% of children struggling with myelomeningocele, aged 6 or more dealing with fecal incontinence, claimed that this condition influenced heavily their social activities and quality of life [6]. In another study it is described that patients, having even only a slight improvement in urinary continence, acquired greater independence, improving the quality of life [7].

So, reducing more and more the frequency of incontinence is a main task in the nursing assistance. Through the survey destined to people struggling with spina bifida, it was possible to observe their knowledge on the problems related to the management of the bladder and bowel apparatus.

The aim of this survey is to improve the nursing assistance of the person with spina bifida to educate them to manage themselves; to understand the level of education on how to manage incontinence and retention, and the problems related to them, and to understand.

## Materials and methods

This analysis was made in collaboration with ASBI (Associazione Spina Bifida Italia), which is the national Italian association founded in 1989 as GASBER (Genitori Associati Spina Bifida Emilia-Romagna) that later in time became the nowadays ASBI to enlarge its scope of influence, in order to prevent the spina bifida and to guarantee a better life to the people struggling with this and their families. The participants of the survey are members of the said association dealing with spina bifida that could freely and anonymously join this analysis with no criteria of exclusion.

The survey was done by using the “Google Docs” platform, then was sent to the members of the association with the help of the president of the association herself, Maria Cristina Dieci.

(https://docs.google.com/forms/d/e/1FAIpQLSdS8xfNJ4fOULG7QD7gDSzgFq4zf5KBLAjbZsNaL9xtKC64XQ/viewform?usp=sf_link).

The clinical and demographic data was computed, such as the age, gender, region of origin, level of deambulation and, in particular, the analysis was focused on the fecal and urinary incontinence.

## Results

### Demographics

**Fig. 1:**
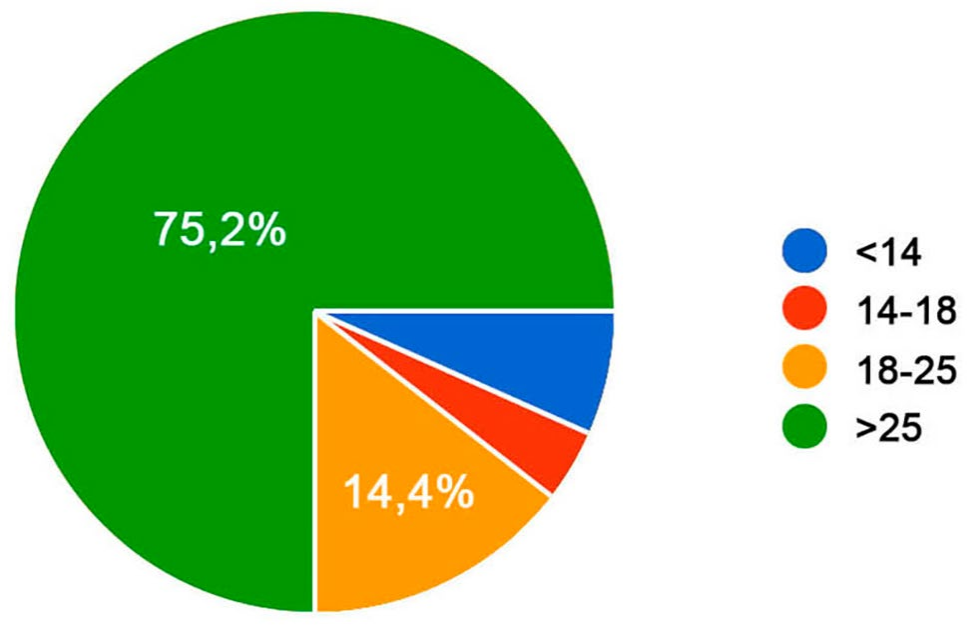
Participants’ age. Age distribution of the participants; Legend: The age registered from the sample is: 6,4% is younger than 14 years old, the 4% is aged between 14 and 18 years old, the 14,4% is aged between 18 and 25 years old and the 75,2% is 25 years old or older

**Fig. 2:**
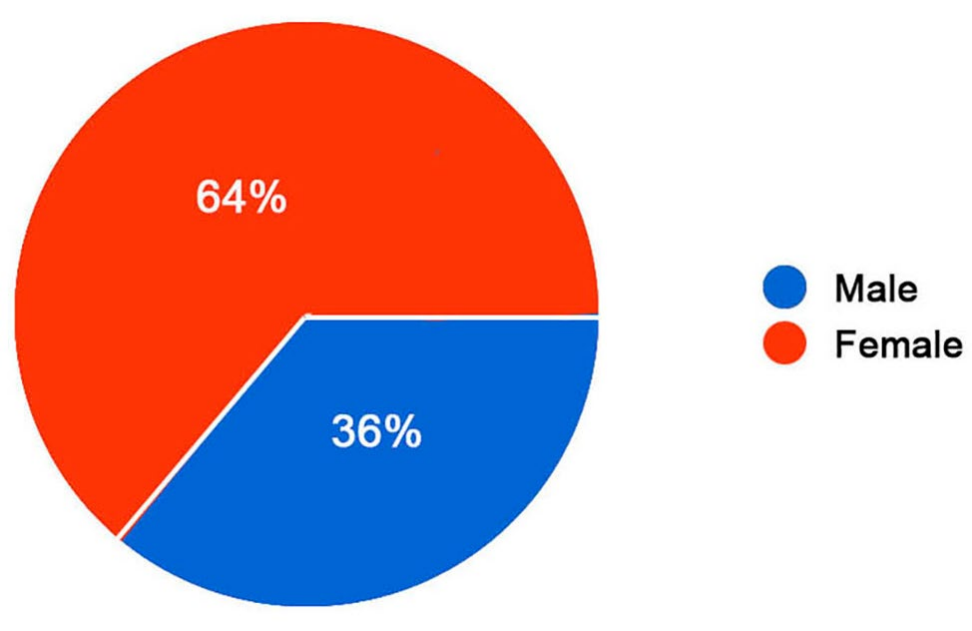
Participants’ gender. Sex gender of the participants. Legend: Among 125 participants, 80 were females and 45 were males

125 participants filed the survey, 80 of them being females and 45 males. The age registered from the sample is: 6,4% is younger than 14 years old, the 4% is aged between 14 and 18 years old, the 14,4% is aged between 18 and 25 years old and the 75,2% is 25 years old or older (Figs. 1 and 2).

Since ASBI is a nation association, the region of origin was asked in order to estimate approximately the diffusion of the condition and the knowledge on the management of the complications analyzed in the survey. The 48,8% of the sample lives in Northern Italy, the 35,20% lives in Southern Italy while the remaining 8% lives in the Centre and isles.

### Deambulation level

**Fig. 3:**
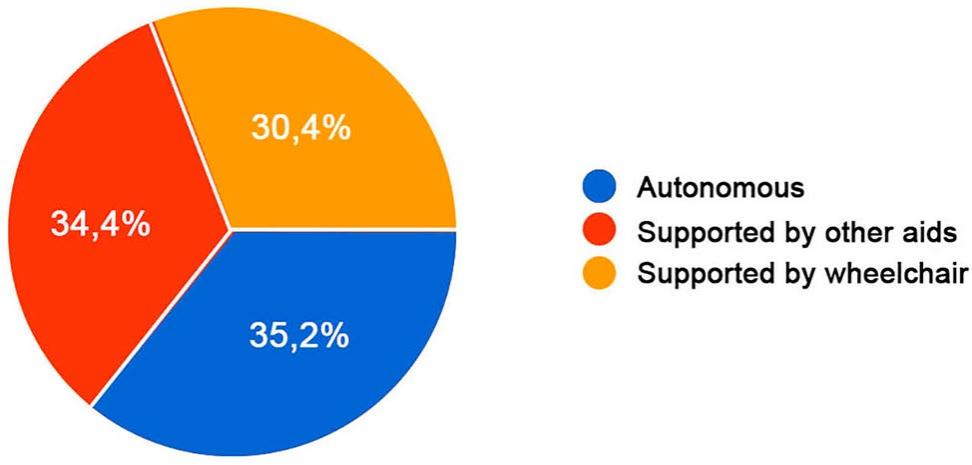
Deambulation level. Level of the autonomy in deambulation; Legend: The 35,2% of the participants claimed to be autonomous with walking, while the 30,4% uses tools such as a wheelchair and the 34,4% is supported by other aids

The 35,2% claimed to be autonomous with walking, while the 30,4% uses tools such as a wheelchair and the 34,4% is supported by other aids, them being asked which kind (Fig. 3).

Thanks to this data, both the level of autonomy and the consequential ability of self-management in daily activities of the subject can be observed.

### Urinary incontinence

**Fig. 4:**
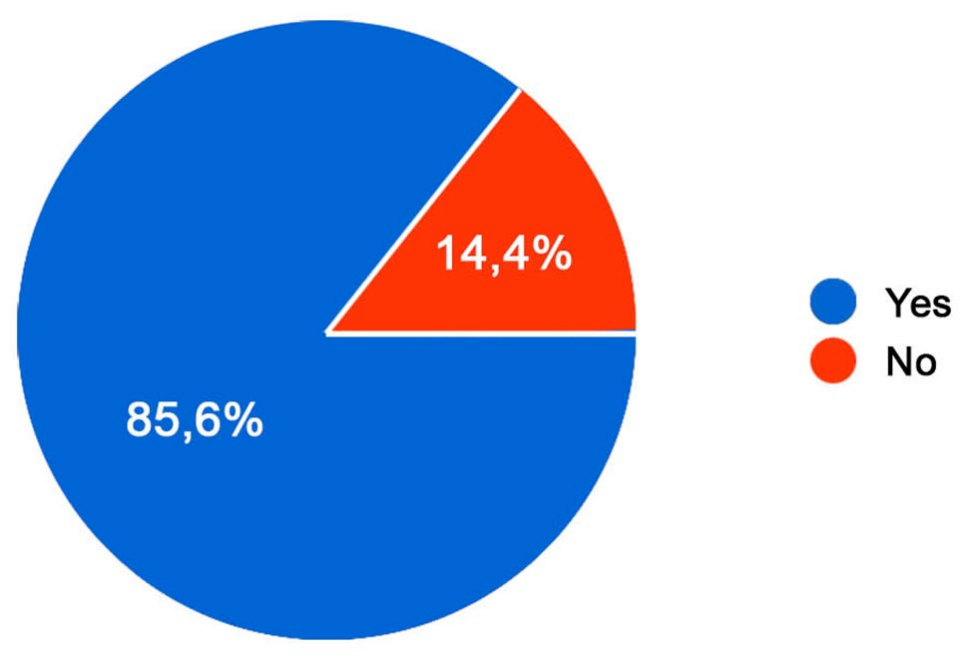
Education received to practice self-catheterization. Awareness of the participants in having had a proper education in performing self-urinary catheterization; Legend: In relation to urinary incontinence, the main aim of this study is to understand if people dealing with spina bifida had received a proper education to practice self-catheterization: the 85,6% claimed they had, unlike the remaining 14,4%

In relation to urinary incontinence, the main aim of this study is to understand if people dealing with spina bifida had received a proper education to practice self-catheterization: the 85,6% claimed they had, unlike the remaining 14,4% (Fig. 4).

**Fig. 5:**
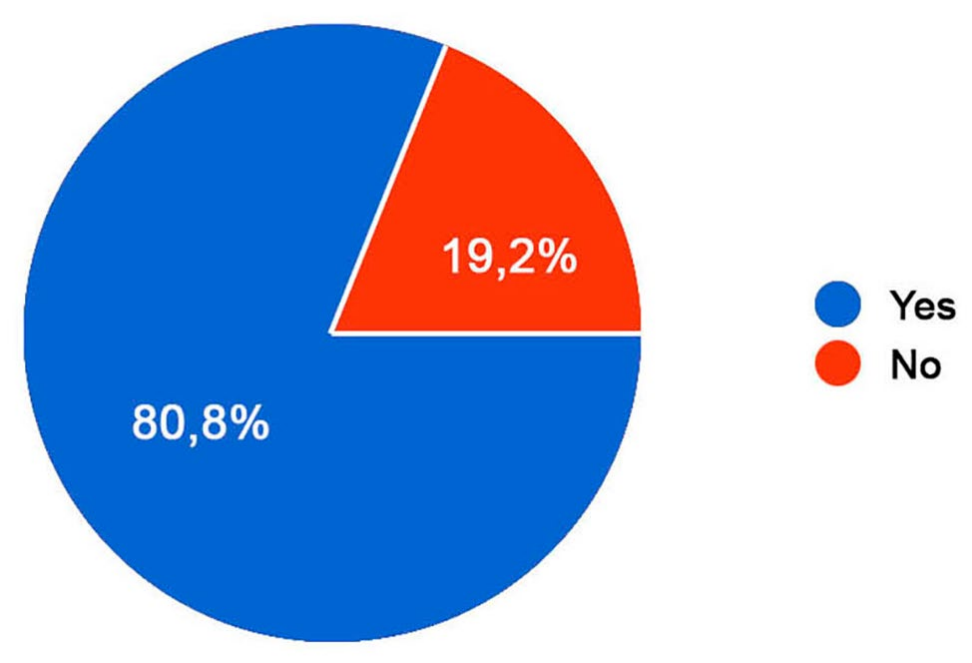
Autonomy to practice self-catheterization. Awareness of the autonomy in performing self-urinary catheterization; Legend: The 80,8% believes to be autonomous in performing auto-catheterization, unlike the remaining 19,2%

Then, the question about “who taught them to perform the technique” was asked, with the 65,2% answering the medical staff while the 34,8% answered the parents. The 80,8% believes to be autonomous in performing auto-catheterization, unlike the remaining 19,2% (Fig. 5).

The question on whether bladder incontinence or retention could in any way negatively affect the quality of life was asked: almost the 59,2% claimed not to feel restrained by their condition of bladder incontinence and retention, unlike the remaining 40,8%. Those who answered positively were asked to specify how.

### Fecal constipation and incontinence

With regards to constipation and incontinence, the 57,6% claimed to struggle with incontinence, the 12% don’t and the 30,4% struggles with both incontinence and constipation.

The 56,8% of the sample claimed to follow and high-fiber diet (fruit, vegetables…) to keep the feces with the right consistency, unlike the 43,2% that doesn’t take enough.

Other than a change in their diet, the question on whether they know how to act in case of constipation was asked: the 88% responded positively, unlike the other 12% that doesn’t know how to act. For those who claimed to know how to intervene, the modality was asked: the 56,6% with mechanical methods (enema, bowel irrigation), 22,1% through medicines and the 21,2% by drinking herbal teas etc.

**Fig. 6:**
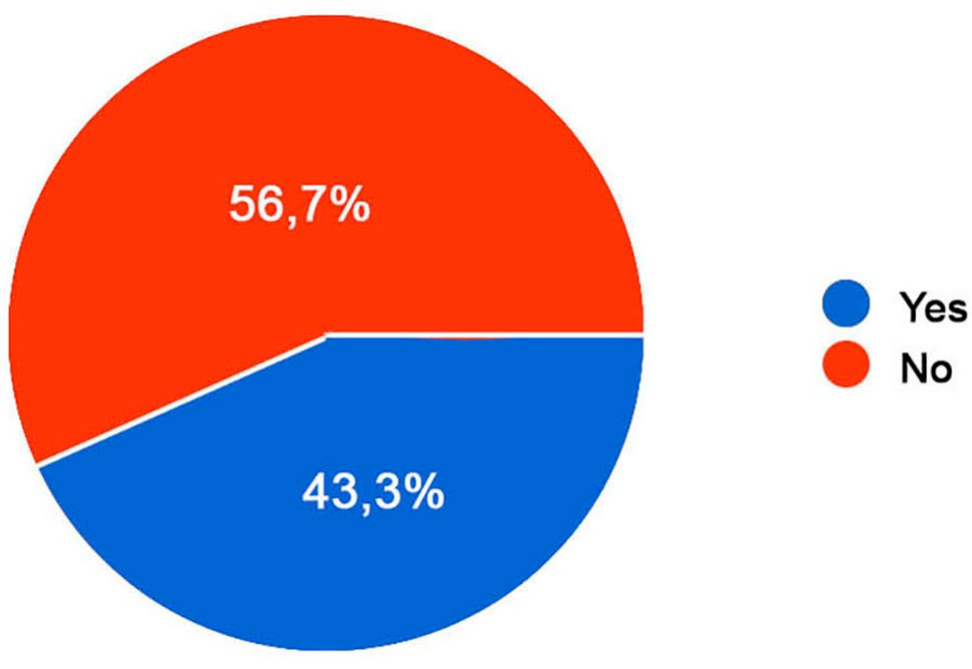
Autonomy to practice rectal irrigation. Autonomy in performing self-rectal irrigation. Legend: 56,7% of the participants feel autonomous in performing rectal irrigation, unlike the remaining 43,3%

Of the patients performing bowel irrigation, the 59,8% was trained to perform the technique unlike the remaining 40,2%. The 81,2% was trained by the medical staff, while the 18,8% learnt with the support of their parents. Obviously, in order to reach independence, it is necessary that the people feel autonomous in performing rectal irrigation, but the majority of them (56,7%) doesn’t feel comfortable enough (Fig. 6). For this reason, we asked if the condition of constipation or fecal incontinence had ever had any negative impact in any way: the 64,2% feels limited unlike the remaining 35,8%.

## Discussion

The survey allows us to have a truthful view on one of the major problems that incur in the life of people dealing with spina bifida. The data collected shows how the urinary and fecal incontinence and constipation affect the day of these people. The high prevalence of incontinence observed is in line with the data reported for children and adolescents signed in the United States Spina Bifida Patient Registry (NSBPR): the 58% struggle with urinary incontinence and the 50% with fecal incontinence [8]. These problems lead the lives of the people with spina bifida, and so, the education is crucial and fundamental. Most of these people was educated by the medical staff on how to perform the self-catheterization technique and the 34,8% only was trained by their parents. The self-catheterization is a fundamental technique to manage bladder incontinence and obtain a greater independence, limiting unpleasant situations such as discharge as much as possible [9]. A recent study observed that the impact of incontinence on quality of life was noted only starting at 10 years old and increases in adolescence, when it corresponds to the discomfort reported by adults [5]. This suggests that the impact on the quality of life may be age-dependent phenomenon.

Since self-catheterization, and potentially other self-care activities, begin at about 9 years old, it is plausible that an increasingly more independent child exposed to more peer interactions in school, could live a condition of diversity as opposed to other children and so affect the quality of their life [10].

It is fundamental that the parent of a children dealing with spina bifida is capable of educating them into practicing self-catheterization, even if it takes time; it is important to encourage the child to actively take part into empty the bladder as soon as possible. Catheterization will then become a natural step if the child starts practicing at an early age, as a 2018 study shows complications (infections, reflux.) and improve quality of life [11]. It is important to find tricks that help the child remember when to perform self-catheterization in specific time intervals, for example it can be done in relation to other activities (meals, snacks) that respects these intervals of time, usually no more than every 6–7 h or one could use reminders on their phone. The child must take the responsibility of empty the bladder; this will contribute on gaining more confidence and autonomy in life. Of course, the training of the parents must be done by the medical staff, which has to be available to fix any issue that might come up in the first phases of the practice. Struggling with urinary incontinence can be embarrassing and isolating, and this can impact on physical and mental health, having a negative impact on the quality of life. There are proofs that testify how urinary and fecal incontinence not only interfere with the daily activities but they’re also associated to a lower self-confidence and participation in social activities as well [12]. The nursing staff not only has to teach how to perform self-catheterization, but it also has to educate the people affected with spina bifida on how to follow an appropriate diet, because by doing so they could improve some issues linked to incontinence and the well-being of the person. The survey shows that only a small part, the 8%, actually drinks 2 L of water a day, the 13,6% drinks 0.5 L, the 36,8% drinks 1 L while the remaining 41,6% drinks 1.5 L. Contrary to belief (just as one of the interviewed claimed), drinking little water isn’t really an effective fix for urinary incontinence. That is because the body requires the right amount of liquids to keep hydrated and to not face dehydration. Furthermore, drinking little water leads to more concentrated urine, which can favor the proliferation of bacteria and create unpleasant infections that might worsen the problem of incontinence. Drinking enough is then so important: it is suggested to drink at least a liter and a half per day, which equals eight cups of water. Another recommendation is to eat healthy, eating a lot of fruit and vegetables as well, and be careful not to exceed with certain foods and drinks such as chocolate, tea, coffee, spicy foods and alcohol, as they actually irritate the bladder walls and can worsen urinary discharge.

We’ve already seen that people dealing with spina bifida show a neurogenic bowel, which is a colon dysfunction (that implies constipation, fecal incontinence, and problems with defecations) due to the loss of the sensory and motor control, or both. With regards to the prevention for fecal incontinence, we can intervene by educating the patients into being careful to certain conditions that may favor it: in the case of constipation, one might need to add fiber into their diet and an adequate amount of water, more than 2 L; in the case of soft or liquid stools (which can easily cause incontinence) it is recommended to reduce or, sometimes, avoid foods or drinks that can irritate the bowel such as alcohol, caffeine, species, spicy and smoky foods which can flame the bowel. From the results showed in the survey, we can observe the aspects on which the nursing staff has to intervene for the management and education of people dealing with spina bifida from a very young age, with the aim to reaching a condition of autonomy and independence in adulthood. Transanal irrigation or colon retrograde irrigation (TAI) is a practice used to assist the feces evacuation from the bowel through the introduction of water in the colon through the anus. In patients dealing with constipation of fecal incontinence, the regular use of transanal irrigation can help stabilize the proper bowel functionalities. This helps the people develop an adequate management of their bowel functions, setting and choosing the right place and time for evacuation. As it was already noted, the 56,7% claims not to be autonomous when it comes to rectal irrigation, and this is something that needs to be taken care of, with a bigger intervention from the sanitary staff and the patients and their families. This is fundamental for the people dealing with spina bifida because, by reducing as much as possible the probability of discharge and/or constipation, leads to them feeling much more self-confident into participating in social life and build social relationship, improving the quality of life. As other studies suggest [13], many participants (almost 50%) were heavily dependent on their caregivers for the management and the support. This information has implications for the future disposal of clinical services to improve the results reached by the patients. A comprehensive ID ambulatory care continuum model should be able to facilitate the management and treatment of common clinical issues, such as urinary tract infections (UTI), pressure areas, neurogenic bowel, sexual dysfunction, and secondary conditions resulting from “overuse” syndromes and “ageing” with disability [14]. This study has some potential limitations: first, selection bias cannot be ruled out, as participants were a selective cohort of people belonging to ASBI that decided autonomously to file the survey anonymously, limiting the possibility of generalizing the results. We acknowledge that other factors may have impacted bowel/bladder and psychological issues in participants and were not studied. However, patient self-report and care report were considered. Important outcomes, such as impact on careers and families and analysis of costs associated with are, were beyond the scope of this study.

## Conclusions

Spina bifida is an example of a chronic pathology that affects a person from birth until death. The management, assistance and prevention of the problems that affect people with spina bifida, whether of a neurological nature (risk of meningeal infections, epilepsy, hydrocephalus) or related to the management of sphincters (incontinence/urinary and fecal retention) or related to the person in his being, engage the nursing staff at 360° from the first years of life. Even when the conditions start to stabilize, one again the figure of the nurse continues to be relevant to support and accompany the person affected through the different stages of life, just like the answers to the survey clearly showed.

The desire of the person with spina bifida is to have a significantly higher level of comfort and normalcy in life and, of course, to have a medical assistance through education, specific technical intervention and psychological support. The progresses done in the last few decades in the field of assistance have improved the condition of life of these people. Nevertheless, the chronicity of this pathology has required and will require more steps and efforts to make their lives more comfortable, therefore it is important to “leave these people behind” but to help them through their path. This way, the figure of the nurse represents a security point, something one can hold to not only for practical support but emotional support as well, to face any difficulty that one may face in their life.

### Electronic supplementary material

Below is the link to the electronic supplementary material.

Supplementary Material 1

## Data Availability

The datasets used and/or analyzed during the current study are available from the corresponding author on reasonable request.

## Abbreviations

UI: urinary incontinence
FI: fecal incontinence
ASBI: Associazione Spina Bifida Italia
GASBER: Genitori Associati Spina Bifida Emilia-Romagna
NSBPR: United States Spina Bifida Patient Registry
TAI: Transanal irrigation
UTI: urinary tract infections
