# Supplementary material for: Nursing management of patients dealing with spina bifida: from the prenatal diagnosis to adulthood / nursing intervention for the improvement of the impact of urinary and fecal incontinence on the quality of life of people dealing with spina bifida
Source: Ital J Pediatr. 2024 Mar 13;50:48. doi: 10.1186/s13052-024-01579-z (PMC10936101; doi:10.1186/s13052-024-01579-z)
Supplement: Supplementary file 1 — Supplementary Material 1 [file 13052_2024_1579_MOESM1_ESM.pdf]

Anna Maria Iannicelli

Department of Translational Medical Sciences,
University of Naples "Federico II",
Via Pansini 5, 80131, Naples, Italy.
Mail: annamaria.iannicelli@unina.it
Tel. +39 0817464559

To
the Editor of International Journal of Pediatrics

March 20, 2023

Dear Editor,

I am pleased to submit an original article entitled "NURSING MANAGMENT OF PATIENTS DEALING WITH SPINA BIFIDA: FROM THE PRENATAL DIAGNOSIS TO ADULTHOOD / NURSING INTERVENTION FOR THE IMPROVEMENT OF THE IMPACT OF URINARY AND FECAL INCONTINENCY ON THE QUALITY OF LIFE OF PEOPLE DEALING WITH SPINA BIFIDA.". This is a survey conducted on 125 people affected by spina bifida, belonging to the ASBI (Associazione Spina Bifida Italia) about the bowel and bladder management.

Regarding the questions of level of deambulation the 35,2% of respondents was autonomous, the 30,4% use wheelchairs while the 34,4% was aid-supported; Regarding urinary incontinence, the 80,8%

reported to be autonomous in performing self-catheterization, while the remaining 19,2% no;

regarding the impact of the said incontinence on social life the 59,2% claimed answered to do not feel restrained because of their bladder incontinence or retention, unlike the remaining 40,8%). Lastly, we focused on fecal constipation and incontinence the 57,6% claimed to struggle with incontinence, the 12% claimed they don't and the 30,4% struggles with both conditions), on the ability of the people dealing with this to intervene to prevent unpleasant situations, by using trans-anal irrigation (the 57,6%

doesn't feel autonomous in performing it).

This manuscript is original research, which has not been previously published and has not been

submitted for publication elsewhere. The authors have no conflict of interests to declare.

Thank you for your consideration.

Sincerely,

Conflict of interest

All authors declare no conflicts of interest in this paper.
